# SHOULD LIMIT VALUES BE SET FOR INFRASOUND CAUSED BY WIND TURBINES?

**DOI:** 10.13075/ijomeh.1896.02422

**Published:** 2025

**Authors:** Małgorzata Pawlaczyk-Łuszczyńska, Tadeusz Wszołek, Adam Dudarewicz, Paweł Małecki

**Affiliations:** 1 Nofer Institute of Occupational Medicine, Department of Vibroacoustic Hazards, Łódź, Poland; 2 AGH University of Science and Technology, Faculty of Mechanical Engineering and Robotics, Department of Mechanics and Vibroacoustics, Kraków, Poland

**Keywords:** infrasound, wind turbines, annoyance, limit values, hearing threshold, environmental exposure

## Abstract

The study focuses on setting outdoor exposure limits for wind turbine infrasound, as most countries currently have no specific limits for this type of noise. A review of the literature on the effects of wind turbine infrasound and the methods used worldwide to measure and assess environmental exposure to infrasound formed the basis for setting limits. According to the literature, human tolerance to infrasound is defined by the hearing threshold, which is not yet standardized. Therefore, a G96 curve (corresponding to tones with the G-weighted sound pressure level (SPL) equal to 96 dB) was used to determine the mean hearing threshold in the 1–20 Hz frequency range. Infrasound that cannot be heard (or felt) is not annoying and does not cause other adverse health effects. The infrasound levels measured around wind farms are well below the hearing threshold. Few countries have set limits for infrasound in either outdoor or indoor environments. The study proposes the G-weighted equivalent SPL as the basis for assessing exposure to infrasound from wind turbines. It also specifies preliminary short-term indices (i.e., G-weighted equivalent SPLs for daytime [L_Geq, D_] and nighttime [L_Geq, N_]) and long-term indices (i.e., averaged G-weighted day-evening-night infrasound level [L_DEN(G)_] and G-weighted night infrasound level [L_N(G)_]). In order to avoid annoyance and other possible harmful effects, regardless of land use, 90 dB was provisionally adopted as an acceptable value for L_Geq, D_ and L_DEN(G)_, and 85 dB for L_Geq, N_ and L_N(G)_. The study highlights the importance of considering specific exposure limits for wind turbine infrasound to ensure the well-being and comfort of people living near wind turbines.

## INTRODUCTION

From the physical point of view, infrasound is defined as an acoustic wave in the frequency range of 0.1–20 Hz [1], while the international standard ISO 7196:1995 defines infrasound as sound or noise with a frequency spectrum in the range of 1–20 Hz [2]. The infrasound frequencies have often been misleadingly described as inaudible. However, the ability to hear infrasound was described by von Bekesy in the 1930s [3]. Low-frequency noise (LFN), on the other hand, refers to noise whose spectrum includes both infrasound (<20 Hz) and low audible (≥20 Hz) components [4]. There is no international definition of LFN, while the Polish standard PN-B-02151-2:2018-01 considers it as broadband noise with the dominant content of frequencies ≤250 Hz [5]. Due to its long wavelength, LFN including infrasound, can propagate over long distances and is almost unaffected by screens and other shadowing areas [1].

Both infrasound and LFN, which occur in the environment may be of natural and artificial origin [1,4]. Examples of natural sources are winds, storms, waterfalls, earthquakes, volcanic eruptions, etc. The most powerful artificial sources of infrasonic waves were associated with nuclear test explosions in the atmosphere, which occurred frequently in the 1950s and 1960s. On the other hand, the most common sources are means of transport, e.g., passenger cars, lorries, ships, trains, helicopters and jet planes. They are also generated by some industrial machinery, including compressors, combustion processes, blast furnaces, boilers, fans, stationary diesel engines, large vibrating surfaces as well as wind turbines [1,4].

Wind turbines are a specific type of noise source that affects large areas. The noise emitted by wind turbines does not resemble ordinary industrial noise. It has unique acoustic characteristics, such as a spectral dominance of LFN, including infrasound as well as amplitude modulation (AM) and tonality [6–8]. In particular, infrasound generated by blade-tower interaction [8,9] is a major source of controversy due to uncertainties about whether infrasound has negative effects on humans.

According to the results of previous studies [10–13], the infrasound levels measured in the vicinity of wind farms (at a distance of 100–500 m from the nearest wind turbine) are significantly (approx. 15–20 dB) below the hearing threshold of infrasound. At some wind farms, tones are modulated at the blade-pass frequency to produce AM tones, which span the infrasound and low-frequency ranges. These AM tones are often perceived as “rumbling” and are audible at distances ≤4 km [8].

With the development of wind energy, it has been suggested that LFN, particularly infrasound, is responsible for adverse health effects in people living near wind farms. The main objective of this study was therefore to answer the question of whether limit values should be set for infrasound from wind turbines and, if so, to propose preliminary outdoor exposure criteria for infrasound specific to wind turbines.

## METHODS

Proposals for acceptable levels of infrasound in the environment have essentially been developed based on a review of:

– recent research on the effects of infrasound from wind turbines, with particular emphasis on hearing thresholds for frequencies <20 Hz,
– methods used worldwide to measure and assess environmental exposure to infrasound.

The evidence review for this study considered the most recent available research, focusing primarily on the topic of “wind turbine infrasound.” The evidence review strategy ensured that eligible evidence was primarily derived from studies published in reputable, peer-reviewed journals.

## RESULTS

### Perception of infrasound and its impact on humans

As mentioned earlier, it has been commonly assumed that infrasound is inaudible. However, early as the 1930s, research showed that if the level was sufficiently high, humans could perceive infrasound. At levels above the hearing threshold, it is possible to feel vibrations in various parts of the body [3]. Slightly above the threshold of auditory perception, infrasound becomes annoying. Its annoyance increases significantly with increasing sound pressure level (SPL) [14]. Furthermore, according to the results of previous investigations, a person's tolerance to infrasound is determined by the threshold of hearing. Infrasound that cannot be heard or felt is not annoying and does not cause other adverse health effects [1,15].

Recent experimental studies on the effects of infrasound generally have mostly focused on brain activity in response to infrasound, often compared to other sounds, including low frequencies [16–21]. They show that infrasound is processed in the auditory cortex, where normal sounds are also processed. Hearing thresholds determined based on brain activity are consistent with those based on classical psychoacoustics. Their results largely confirm previous observations. There is no evidence that infrasound at SPLs well below their hearing threshold can affect human health and well-being [22,23].

There are 2 opposing views on the potential effects of infrasound associated with the operation of wind turbines [24]. According to the first, infrasound causes adverse physiological and psychological effects in humans even if it is not heard or felt. In contrast, the second option suggests that modern wind turbines generate infrasound at levels well below the threshold of auditory perception and are unlikely to cause adverse effects in humans.

Hypotheses about the adverse effects of exposure to inaudible infrasound include, among others, vibroacoustic disease (VAD) and wind turbine syndrome (WTS) [25–27]. Vibroacoustic disease is associated with abnormal growth of extracellular matrices (collagen and elastin) in the absence of an inflammatory process, as well as depression, irritability and cognitive impairment. It was initially associated with long-term (≥10 years) occupational exposure to LFN (≤500 Hz) at high SPLs (≥90 dB) [25]. Later, due to the ubiquity of LFN, the occurrence of VAD in people exposed to low-level infrasound from wind turbines was suggested [26]. In turn, people suffering from WTS reported serious symptoms, including insomnia, headaches, tinnitus, dizziness, nausea, panic attacks, and heart palpitations, that developed after wind turbines were erected near their homes. According to Pierpont [27], these symptoms are caused by LFN, including infrasound and vibrations from wind turbines, which affect the body's balance system.

Opponents argue that these symptoms have a psychological basis and are attributable to the nocebo effect [28]. It has been shown that expectations about the health effects of infrasound exposure, rather than the level of exposure, determine the extent and intensity of symptoms. Negative expectations increase symptoms and positive expectations decrease symptoms and improve mood [28].

There is still no irrefutable evidence linking WTS and VAD to exposure to infrasound associated with wind turbine operation [22]. Such conclusions can be drawn from a recent comprehensive and carefully controlled experimental study by Marshall et al. [29], which was focused on the health effects of infrasound exposure. This confirmed the absence of any physiological and psychological disturbances after 72 h of exposure to infrasound levels below the hearing threshold [29]. On the other hand, Zajamšek et al. [30] investigated the detectability of wind turbine infrasound and concluded that participants, including a subgroup of people who self-reported suffering from sleep disturbance due to wind turbine noise, were unable to detect the presence or absence of wind turbine infrasound above chance when exposed to typical SPLs [30]. At the same time, however, the latter study also showed that sub-audible infrasound interferes with the auditory perception of higher-frequency noise, supporting the need for further research to understand the mechanisms underlying infrasound perception and how infrasound affects the perception of audio-frequency stimuli [30].

The results of previous research have also shown that infrasound at the levels occurring in the vicinity of wind turbines does not affect perception, annoyance or autonomic nervous system responses [22,23,31]. Furthermore, a review of the literature by Baliatsas et al. [32] concludes that there is no evidence that LFN, in particular infrasound, can cause adverse health effects other than those caused by higher-frequency noise.

In turn, a Finnish research team [33–35] published the results of an extensive cross-sectional and laboratory study to analyze the potential effects of exposure to infrasound from wind farms. Long-term recording of infrasound levels, along with comprehensive community surveys, were conducted in areas where possible symptoms of negative effects from nearby wind farms had been reported [33,34]. Residents of these areas also took part in laboratory tests, during which they were exposed to the highest levels of infrasound that had been recorded in the field [35]. The subjects were divided into 2 groups (“symptomatic” and “non symptomatic”) depending on the reported or unreported negative effects of infrasound.

The results showed that 5% of respondents (15% of those living within 2.5 km from the closest wind turbine) reported symptoms (e.g., headaches, heart rate variability, sleep disturbances, etc.) that they attributed to wind turbine infrasound [34]. On average, “symptomatic” respondents lived closer to the wind farm than those without symptoms. Symptoms were correlated with the occurrence of chronic diseases, annoyance associated with various visual and auditory aspects of wind turbines (e.g., shadow flicker), and the recognition of these devices as a health hazard. Apart from annoyance and sleep disturbance, there were no consistent associations between exposure to wind turbine noise and reported health problems. It was also found that those reporting symptoms did not show increased sensitivity to infra-sound. Among other things, the presence of infrasound did not affect subjective ratings of annoyance, heart rate and heart rate variability, and skin conductance (physiological measures of stress). No differences were found between the 2 groups [33–35].

Therefore, infrasound generated by wind turbines does not pose a direct threat to human health and well-being because the SPLs are below the threshold of auditory perception, especially since normal pressure changes in the body associated with heartbeat and breathing result in higher levels of infrasound in the inner ear than in the case of wind farms [36]. However, auditory research and complaints about environmental noise indicate that there exists a significant, small subgroup within the population which is sensitive to infrasound and LFN [4].

### Hearing threshold levels of infrasound

The frequency range of 20 Hz − 20 kHz is traditionally accepted as the range of human hearing, and within this range, the so-called “normal hearing thresholds” have been standardized [37–39]. As previously mentioned, infrasound can also be heard at sufficiently high SPLs. Hearing thresholds for these frequencies have not yet been standardized, but attempts have been made by Vercammen [40,41], Møller and Pedersen [3], Kurakata and Mizunami [42], among others.

For example, Vercammen [40] and Moller and Pedersen [3] performed an analysis of hearing thresholds recorded in 1972–1987 and 1967–2001, respectively, and determined mean hearing thresholds <20 Hz. In both cases, the mean hearing threshold was fitted to a straight line [40,41], while Møller and Pedersen [3] also fitted the analyzed data to a second-order polynomial regression curve. On the other hand, Kurakata and Mizunami [42] determined a statistical distribution of infrasound hearing thresholds for young otologically normal subjects.

The line fitted to the average hearing threshold has a similar slope (12 dB/octave, 1–20 Hz) to that of the G-weighting characteristics for infrasound [2]. This corresponds to tones with a G-weighted SPL equal to 96 dB and is called the G96 curve. The average hearing threshold for the 10 Hz reference tone is approx. 96 dB, while for the 2 Hz and 16 Hz tones it is 124 and 88 dB, respectively. In the graph, the average hearing threshold of infrasound can be presented by a straight line with a slope of 12 dB/octave in the frequency range of 1–20 Hz and a SPL of 96 dB at 10 Hz. The G96 curve can also be described using the formula:



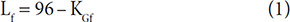



where:

L_f_ – the SPL in the 1/3-octave band with center frequency f, in dB,

K_Gf_ – the relative response of the G-weighting characteristics in the 1/3-octave band with center frequency f, in dB.

**Figure 1.**
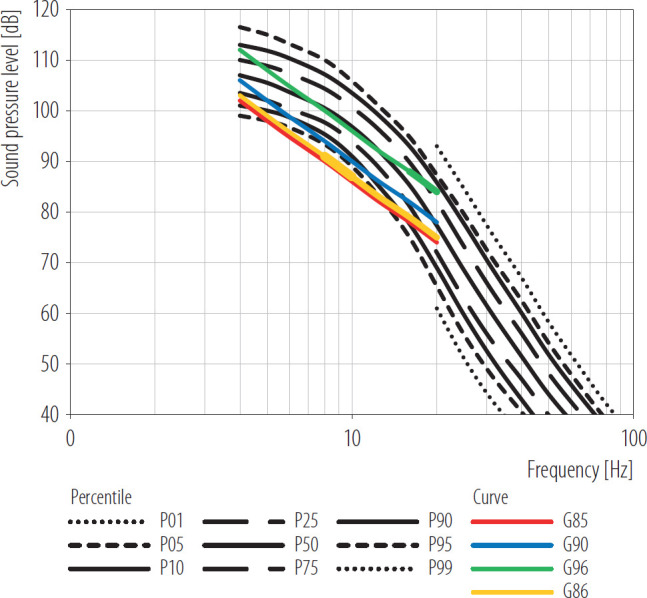
Statistical distribution of infrasound hearing thresholds (from the 5th percentile [P05] to the 95th percentile [P95]) for young (aged 18–25 years) otologically normal subjects as determined by Kurakata and Mizunami [42] and the G96, G90, G86 and G85 curves [3,40,41,42]

Considering the variability of hearing thresholds observed in various experiments (standard deviation of about 5 dB on average), the G86 curve was taken as the threshold for hearing infrasound exceeded by 90–95% of the population [40]. The aforementioned G96 and G86 curves, along with some other proposed hearing thresholds in the infrasonic frequency range, are shown in Table 1 and Figure 1.

**Table 1.** Mean infrasound hearing threshold determined for otologically normal young adults (aged 18–25 years) along with the G96 and G86 curves, based on world studies conducted in 1967–2001 [3,40,41]

Frequency	Hearing threshold [3] [dB](M)	Sound pressure level [dB]	
		G86 curve [40]	G96 curve [41]
1Hz		129	139
1.25 Hz	133.5	123.5	133.5
1.6 Hz	128.6	118.6	128.6
2 Hz	124.3	114.3	124.3
2.5 Hz	120.1	110.1	120.1
3.15 Hz	116	106	116
4 Hz	112	102	112
5 Hz	108	98	108
6.3 Hz	104	94	104
8 Hz	100	90	100
10 Hz	96	86	96
12.5 Hz	92	82	92
16 Hz	88.3	78.3	88.3

The distribution of the infrasound hearing thresholds determined by Kurkata and Mizunami [42] (Figure 1) indicates that 10% of young people can hear a tone of a frequency of 10 Hz at approx. 90 dB. Furthermore, an analysis of the hearing thresholds among young people (around 20 years old) and older individuals (>60 years old) revealed that the difference in their medians, regardless of frequency, is about 10 dB [43]. This means that, in contrast to their often significantly reduced sensitivity at high frequencies, older people retain good hearing at low frequencies (i.e., they are slightly less sensitive to infra-sound) [43].

### Review of existing evaluation criteria for infrasound and low-frequency noise

The international standard ISO 7196:1995 [2] recommends the use of G-weighting frequency characteristics for the assessment of infrasound. As previously mentioned, results of laboratory studies have shown that the subjective assessment of annoyance is strongly correlated with the equivalent G-weighted SPL [14].

To date, only a few countries, such as Denmark, Australia and Japan, have established permissible indoor infra-sound levels, but these do not directly apply to wind turbines [44]. In Denmark, for example, the recommended permissible infrasound levels inside residential buildings during the day, evening, and night, as well as in classrooms and offices, are 85 dB, while in commercial premises, they are 90 dB, with a 5 dB penalty for impulsive noise [45]. The limits adopted in Australia are modelled on those used in Denmark [46]. In Japan, it is believed that if the measured G-weighted SPL ≥92 dB, it is very likely that infra-sound has an effect [47].

Limit values for outdoor LFN have been established in some states and provinces of Australia and Canada, as well as in Japan, but they do not apply directly to wind turbines [44]. Some assume that a difference >20 dB between C- and A-weighted SPLs indicates the presence of LFN, setting the C-weighted equivalent SPL limit values at 65 dB for day and 60 dB for night. The LFN criteria for dwellings are in use in some European countries [48]. Evaluation of exposure to LFN is usually based on the frequency analysis in 1/3-octave bands in various frequency ranges 8–250 Hz (Table 2). In most cases, the measured SPLs are compared with corresponding reference curves. Only in Denmark and Germany are the results of the frequency analysis subjected to further calculations taking into account the tonal and/or impulsive character of the noise [48].

**Table 2.** Evaluation criteria concerning indoor exposure to low-frequency noise used in some European countries and Japan (based on Pawlaczyk-Łuszczyńska and Dudarewicz [48])

1/3-octave frequency band	Permissible sound pressure levels in 1/3-octave bands [dB]							
	Germany	Sweden	Netherlands	Great Britain	Poland	Finland	Japan	
							*	**
5 Hz	—	—	—	—	—		70	—
6.3 Hz	—	—	—	—	—		71	—
8 Hz	103^+s/o^***	—	—	—	—		72	—
10 Hz	95^+5/0^	—	—	92	80.4		73	90
12.5 Hz	87^+5/0^	—	—	87	73.4		75	88
16 Hz	79^+5/0^	—	—	83	66.7		77	83
20 Hz	71^+5/0^	—	74	74	60.5	74	80	76
25 Hz	63^+5/0^	—	64	64	54.7	64	83	70
31.5 Hz	55.5^+5/0^	56	55	56	49.3	55	87	64
40 Hz	48^+5/0^	49	46	49	44.6	46	93	57
50 Hz	40^+5/0^	43	39	43	40.2	49	99	82
63 Hz	33.5^+5/0^	41.5	33	41.5	36.2	44	—	47
80 Hz	28^+10/5^	40	27	40	32.5	42	—	41
100 Hz	23.5^+l5/10^	38	22	38	29.1	40	—	—
125 Hz	—	36	—	36	26.1	38	—	—
160 Hz	—	34	—	34	23.4	36	—	—
200 Hz	—	32	—	—	20,9	34	—	—
250 Hz	—	—	—	—	18,6	32	—	—

* Evaluation criterion in case of complaints due to rattling windows and doors.

** Evaluation criterion in case of complaints due to mental and physical discomfort.

*** Penalty for day/night tonal noise.

In Denmark, for example, a low-frequency A-weighted SPL (L_pA, LF_) is determined based on results obtained in 1/3-octave bands from 10 to 160 Hz. Recommended limits for homes are 25 dB during the day and 20 dB at night time. In offices, classrooms, etc., L_pA, LF_ should not exceed 30 dB, and in other rooms − 35 dB [45].

In Germany, according to the recommendation of DIN 45680:1997 [49], a difference between the (equivalent or max.) C- and A-weighted SPLs ≥20 dB indicates the occurrence of LFN. The assessment is based on a frequency analysis in the 1/3-octave frequency bands between 10 Hz and 80 Hz. However, in exceptional cases, the 1/3-octave bands of 8 Hz and/or 100 Hz are also considered. If the noise is not tonal the equivalent-continuous A-weighted SPL in the 10−80 Hz frequency range is calculated based only on bands exceeding the hearing threshold. Whereas, for tonal noise, the SPL of the dominant 1/3-octave band (or bands) is compared with the hearing threshold modified by penalty, depending on the frequency and the time of the day [49].

In the Netherlands, several proposals for criteria for assessing LFN have been prepared, including a criterion based on the frequency analysis in the 1/3-octave bands 10–200 Hz as well as on the hearing thresholds of the 10% best-hearing individuals in the unselected 50–60 years age group, taken as reference values [48].

In 1995–1998, the Polish criteria for the assessment of LFN in dwellings were developed [50]. The frequency analysis in the 1/3-octave bands 10–250 Hz was proposed as the basis for the evaluation, and the A10 curve was chosen as the reference curve.

This curve was derived from:



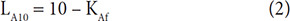



where:

L_A10_ − the SPL in the 1/3-octave band with center frequency f, in dB,

K_Af_ − the relative response of the A-weighting characteristics in the 1/3-octave band with center frequency f, in dB and f of 10–250 Hz.

Later, after some updates, the previously mentioned criteria were published as the Polish standard PN-B-02151-2:2018-2 [5]. Denmark is perhaps the only country so far to have regulations specifying acceptable levels of LFN indoors caused by wind turbines. This is a low-frequency A-weighted SPL of 20 dB [51].

It is worth noting that earlier Poulsen [52] and Subedi et al. [53] compared various criteria used for evaluating LFN in dwellings. For example, Poulsen [52] played different environmental LFNs at relatively low A-weighted SPLs (20−35 dB) to subjects in the laboratory and carried out an analysis to find out that the Danish method gave the best correlation with subjective evaluations, but it depended on the 5 dB penalty for impulsive noise (e.g., from discotheque music). Without this penalty, the Danish method is similar to the Swedish and German (tonal and non-tonal) methods.

Subedi et al. [53] measured the annoyance of low-frequency pure and combined tones in a laboratory experiment. They compared these results (median values) to the evaluation obtained from three objective methods, namely one based on Moore's loudness model, the total energy summation model, and the low-frequency A-weighted SPL. Among these methods the latter one gave the best correlation. However, the aforesaid parameter cannot be measured directly. This is calculated based on the results of the frequency analysis in the 1/3-octave bands of 10–160 Hz using the following formula:



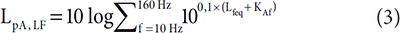



where:

L_pA, LF_ – the low-frequency A-weighted SPL, in dB, L_feq_ is the measured SPL in 1/3-octave frequency bands 10–160 Hz, in dB,

K_Af_ – the relative response of the A-weighting characteristics in 1/3-octave bands of 10–160 Hz, in dB [44].

Considering the simplicity of the measurements, it was finally decided to propose limit values for infrasound only. The higher frequency range of LFN (20–250 Hz) can be covered by C- or/and A-weighted SPL measurements.

### Proposed evaluation criteria for infrasound exposure

Although there is currently no hard evidence that inaudible infrasound affects human health and well-being, preliminary limits for infrasound in the environment from wind turbines have been proposed. Assuming that the G-weighted equivalent SPL (L_Geq, T_) would be the basis for assessing preliminary environmental exposure to infra-sound, similar to that for ordinary noise, the following criteria have been proposed:

– short-term indices, i.e., G-weighted equivalent SPL for daytime (L_Geq, D_) and G-weighted equivalent SPL for nighttime (L_Geq, N_),
– long-term indices, i.e., averaged G-weighted day-evening-night infrasound level (L_DEN(G)_) and G-weighted night infrasound level (L_N(G)_).

The short-term indices are intended to be applied for establishing and controlling the conditions of use of the environment in relation to one day, while the long-term (annual) indices can be used to prepare the environmental noise protection programs. LDEN(G) is calculated using the following formula:



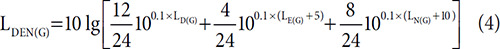



where:

L_D(G)_ – the long-term daily G-weighted infrasound level, in dB, averaged over all days in a year, defined for the time interval from 6:00 a.m. to 6:00 p.m.;

L_E(G)_ – the long-term evening G-weighted infrasound level, in dB, averaged over all evenings in a year, defined as the time interval from 6:00 p.m. to 10:00 p.m.;

L_N(G)_ – long-term night G-weighted infrasound level, in dB, averaged over all nights in a year, defined as the time interval from 10:00 p.m. to 6:00 a.m.

Irrespective of land use, the short-term indices (G-weighted equivalent SPL) were provisionally set at 90 dB for a reference time (T) = 16 h during the day and 85 dB for T = 8 h during the night. Similarly, the acceptable values for the long-term indicators were proposed to be 90 dB for L_DEN(G)_ and 85 dB for L_N(G)_, regardless of land use (Table 3).

**Table 3.** Proposals of permissible levels of infrasound in the environment caused by wind turbines expressed by the indices L_Geq, D_ and L_Geq, N_, which are applicable to the establishment and control of conditions of use of the environment, for one day, as well as the indices L_DEN(G)_ and L_N(G)_, applicable to the conduct of long-term infrasound protection policy

Type of terrain	Permissible level of infrasound in the environment caused by wind turbines [dB]	
	L_Geq, D_^a^/L_DEN(G)_	L_Geq, N_^b^/L_N(G)_
Protection zone of the SPA	90	85
Hospitals areas outside the city	90	85
Areas of single-family housing	90	85
Areas of buildings connected with permanent or temporary residence of children and young people	90	85
Residential care homes	90	85
Urban hospital areas	90	85
Areas of multi-family housing and collective residential buildings	90	85
Areas for farm buildings	90	85
Recreational and leisure areas	90	85
Residential and services areas	90	85

L_DEN(G)_ – the long-time (annual) index expressed as the averaged G-weighted day-evening-night infrasound level (a descriptor of the infrasound level based on the energy mean G-weighted equivalent SPL over a whole day with a 10 dB penalty for night time infrasound [10:00 p.m. – 6:00 a.m.] and an additional 5 dB penalty for evening infrasound [i.e., 6:00 p.m. – 10:00 p.m.], calculated including all days during a year); L_Geq, D_ – the short-term (in relation to one day) index expressed as the G-weighted equivalent SPL during daytime; L_Geq, N_ – the short-term (in relation to one day) index expressed as the G-weighted equivalent SPL during nighttime; L_N(G)_ – the long-term (annual) index expressed as the G-weighted night infrasound level calculated taking into account all nights during a year.

a For reference time interval T = 16 h.

b For reference time interval T = 8 h.

### Justification of setting preliminary limits for infrasound

Despite the recent and rapid development of wind power, the local communities in Poland often oppose new wind turbine projects. The main arguments against wind turbines are the landscape littering and shadow flicker accompanying the operation of wind turbines. Possible adverse and/or annoying effects of noise, especially as LFN and infrasound are a source of particular concern. Thus, the objective of this study was to propose preliminary permissible levels of environmental exposure to infrasound which is specific to wind turbines. The results of some recent epidemiological and experimental studies do not suggest that infrasound from wind turbines is responsible for harming the health and well-being of people living near wind turbines [22,23]. Furthermore, infrasound levels measured in the vicinity of wind farms are not only lower than their hearing threshold, but they are also lower (or comparable to) SPLs caused by some common sources of infrasound [10–13,54]. Moreover, human's tolerance to infrasound is defined by hearing threshold. Infrasound that cannot be heard (or sensed) is not annoying and does not cause adverse health effects [1,15].

For example, infrasound in urban areas can reach G-weighted SPLs of 60–70 dB [54]. It is usually 10 dB higher during the day than at night. Infrasound associated with human activity regularly exceeds 85 dB (especially in the case of traffic or the use of air conditioning). In rural areas, the level of infrasound depends largely on the weather conditions. It varies between 40 dB and 70 dB during periods of low and high wind respectively [54].

While the vast majority of studies confirm that infrasound at levels well below the hearing threshold has no adverse effects, there is also evidence, albeit much less, that infra-sound can adversely affect human well-being [31]. For example, a recent literature review by Flemmer and Flemmer [31] shows that according to the official status of the Australian Senate Committee on Wind Turbines, there is credible evidence from people living near wind turbines complaining of adverse health symptoms. Annoyance and sleep disturbance appear to be the most common symptoms of infrasound, and these symptoms increase as the distance of the residence from the wind turbine decreases. The results of a study by the Council of Canadian Academies, also cited in the above review, confirm that wind turbine infrasound can cause annoyance and sleep disturbance [31]. It was also found that annoyance increased with increasing sound levels and duration of exposure. This suggests that despite infrasound levels below the hearing threshold, some people living near wind farms may become sensitized to infrasound, while others may become accustomed to it. As a result, people sensitive to infra-sound may suffer from symptoms of chronic stress [31].

What is more, according to the results of the recent study by Zajamšek et al. [30], sub-audible infrasound has been shown to interfere with the auditory perception of higher-frequency noise, supporting the need for further research to understand the mechanisms underlying infrasound perception and how infrasound affects the perception of audio-frequency.

Furthermore, the intensive development of wind energy around the world has been accompanied not only by an increase in the number of turbines installed, but also by an increase in their capacity from an average of 100 kW in the 1990s to 2–3 MW at present. Hub heights are currently around 100 m with rotor blades about 50 m long, and 10 MW prototypes exceeding 200 m in height have been developed. The increasing size of turbines has raised concerns that the sound characteristics will shift to lower frequencies [10]. This should be taken seriously, as sounds with prominent infrasound (1–20 Hz) and low-frequency (20–200 Hz) components can affect human health and well-being to a greater extent than sounds without such components.

Based on these considerations, preliminary outdoor permissible levels for wind turbine infrasound have been proposed. The following assumptions were made in setting the above limits. First, it was assumed that the threshold of auditory perception determines a person's tolerance to infrasound and should be taken into account. Second, since infrasound cannot be heard (or felt), is not annoying, and does not cause other adverse effects [1,15], it was assumed that ideally acceptable infrasound levels should be below the average hearing threshold. Third, taking into account the recommendations of the international standard ISO 7196:1995 [2] and the simplicity of measurements, it was decided that the basis for the assessment of environmental exposure to infrasound would be the G-weighted equivalent SPL. Formal requirements for testing laboratories to ensure measurement consistency also indicated the reasonableness of this assumption. It is worth noting that the Polish Central Office of Measures calibrates sound level meters or sound analyzers in the infrasound frequency range for compliance with the requirements of ISO 7196:1995 [2].

As mentioned above, the mean hearing threshold for infrasound is determined by the G96 curve which in turn corresponds to G-weighted SPL equal to 96 dB. It has been assumed that 50% of individuals can perceive infrasound at such SPL [41]. To reduce this percentage, it would be necessary to take into account the variability of hearing thresholds found in previous studies, expressed as the mean (M) of the standard deviation (SD) (equal to approx. 5 dB), and adopt the acceptable value at the lower levels.

This study proposes preliminary permissible G-weighted SPLs of 90 dB and 85 dB, corresponding to the G90 and G85 curves, respectively. These curves are represented in Figure 1 as straight lines with a slope of 12 dB/octave in the 1–20 Hz frequency range and SPLs of 90 dB and 85 dB at 10 Hz. These lines are shifted downward by 6 dB and 11 dB, respectively, in parallel with the G96 curve.

Figure 1 shows, when comparing the G90 and G85 curves with the hearing threshold distribution determined by Kurakata and Mizunami [42], the percentile of people who can hear infrasound varies with frequency. For example, in the case of the G90 curve, this percentile varies from approx. 5% (at 8 Hz) to 50% (at 4 or 20 Hz), while in the case of the G85 curve, it remains within the range from 5% (at 5 or 13 Hz) to about 35% (at 20 Hz).

**Figure 2.**
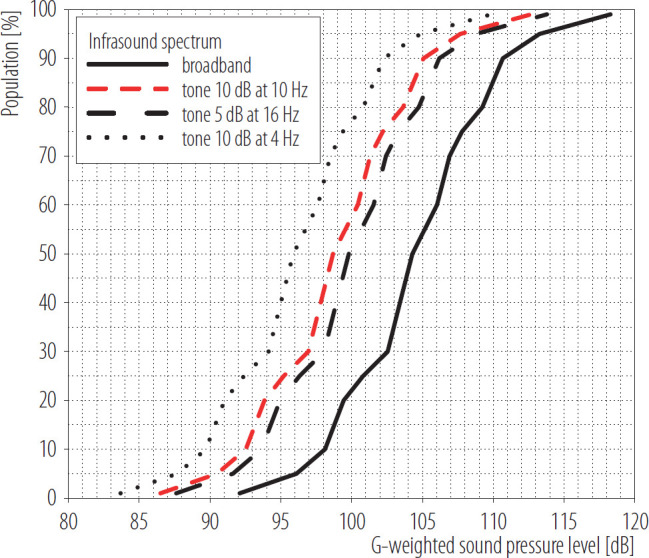
Statistical distribution of the percentage of subjects who may perceive infrasound as a function of the G-weighted sound pressure level determined in the study based on statistical distributions of infrasound hearing thresholds as specified by Kurakata and Mizunami [42]

Assuming that infrasound is audible when at least one 1/3-octave band in the range 4–16 has SPL equal to the selected hearing threshold, the G-weighted SPL corresponding to a given percentile of the hearing threshold distribution can be calculated. By repeating this process for different infrasound spectra and percentiles, it is possible to determine the percentage of individuals who can perceive infrasound at a given G-weighted SPL (Figure 2).

The black line refers to infrasound broadband spectra with a shape according to the hearing thresholds of successive percentiles, while the remaining curves correspond to various tonal infrasound spectra shaped corresponding to the hearing thresholds of successive percentiles lowered by 5 dB or 10 dB, except for the 1/3 octave bands indicated.

Assuming a normal distribution of hearing thresholds (M±SD 96±5 dB) [3], infrasound at the G-weighted SPL of 90 dB may be heard by approx. 11.5% of the population, and in consequence being annoyed. On the other hand, infrasound at a G-weighted SPL of 85 dB can be heard by about 1.5% of the population. Such percentiles of subjects annoyed by infrasound are usually recognized as acceptable from the point of view of setting exposure limits [55]. Similar conclusions can be formulated when analyzing Figure 2.

In summary, given the characteristics of infrasound propagation, the preliminary outdoor infrasound limits proposed in this paper are similar to the indoor limits that have been in use in Denmark and Japan for >20 years.

## CONCLUSIONS

– According to the literature, human tolerance to infra-sound is determined by the hearing threshold, which has not yet been standardized. Therefore, the G96 curve was used to determine average hearing thresholds in the 1–20 Hz frequency range.
– Infrasound levels measured in the vicinity of wind farms are well below the hearing threshold for infrasound.
– It is believed that inaudible (or imperceptible) infra-sound is not annoying and does not cause other adverse health effects.
– While most studies confirm that infrasound at levels well below the hearing threshold has no adverse effects, limited evidence suggests that inaudible infrasound can cause annoyance and sleep disturbance, suggesting that some people living near wind farms may become sensitized to infrasound and suffer chronic stress symptoms. This has led to the proposal of outdoor limits for infra-sound from wind turbines.
– To date, only a few countries have set outdoor or indoor limits for infrasound.
– The method of measurement has been taken into account when setting the limits. Considering the recommendations of ISO 7196:1995 and the simplicity of measurement, L_G_ has been proposed as the basis for assessing exposure to infrasound.
– Preliminary short-term (L_Geq, D_ and L_Geq, N_) and long-term (L_DEN(G)_ and L_N(G)_) indices have been proposed. To avoid annoyance and other possible harmful effects, regardless of land use, 90 dB was adopted as an acceptable value for L_Geq, D_ and L_DEN(G)_, and 85 dB for L_Geq, N_ and L_N(G)_.
– The above proposals do not exclude the possibility of carrying out a frequency analysis in 1/3-octave bands, particularly in relation to the daily use of the environment.
– The rapid global expansion of wind energy is associated not only with an increase in the number of turbines installed, but also with an increase in their capacity, which in turn is associated with an increase in their dimensions. The increase in turbine size has raised concerns about a shift in noise characteristics towards lower frequencies, which should be taken seriously, as noise with significant infrasound components may have a greater impact on human health and well-being than noise without such components.
– The preliminary limits for wind turbine infrasound proposed in this study should be reviewed based on the results of longitudinal epidemiologic studies. Further research is necessary before a firm conclusion can be drawn about the potential effects of wind turbine infra-sound on people living near wind farms.
